# The regulatory role of the circELMOD3-associated ceRNA network in the progression and prognosis of hepatocellular carcinoma

**DOI:** 10.3389/fgene.2025.1521360

**Published:** 2025-04-15

**Authors:** Deyuan Li, Meiliang Liu, Mingshuang Lai, Lijun Wang, Liling Wei, Siqian Wu, Si Liang, Shun Liu, Xiaoyun Zeng

**Affiliations:** ^1^ School of public health, Guangxi Medical University, Nanning, Guangxi, China; ^2^ Guangxi Colleges and Universities Key Laboratory of Prevention and Control of Highly Prevalent Diseases, Guangxi Medical University, Nanning, Guangxi, China; ^3^ Key Laboratory of Early Prevention and Treatment for Regional High Frequency Tumor (Guangxi Medical University), Ministry of Education, Nanning, Guangxi, China; ^4^ Guangxi Key Laboratory of Early Prevention and Treatment for Regional High Frequency Tumor (Guangxi Medical University), Nanning, China; ^5^ Department of Epidemiological and Health Statistics, School of Public Health, Guilin Medical University, Guilin, Guangxi, China

**Keywords:** circELMOD3, hepatocellular carcinoma, prognosis, ceRNA network, cell cycle

## Abstract

**Background:**

Our previously research has validated the effect of circELMOD3 on HCC tumor inhibition. However, further investigations are warranted to investigate the prognostic significance of circELMOD3 in HCC and its regulation via the competitive endogenous RNA (ceRNA) network.

**Methods:**

The gene expression profiles and clinical information were obtained from The Cancer Genome Atlas (TCGA-LIHC) and International Cancer Genome Consortium (ICGC). Base on the circMine, miRWalk and TargetScan database, we constructed circELMOD3-miRNA-mRNA network. Univariate Cox and least absolute shrinkage and selection operator (LASSO) regression analysis was used to constructed the prognostic model. Additionally, Gene set enrichment analysis (GSEA) was conducted for the prognostic-related genes. Finally, the expression levels of genes and proteins were respectively assessed by quantitative real-time polymerase chain reaction (qRT-PCR) and Western blotting.

**Results:**

We constructed a ceRNA network comprising circELMOD3, 5 miRNAs, and 274 mRNAs. From this ceRNA network, we identified four prognostication-relation genes to develop a survival prediction model. In the TCGA-LIHC training set, the area under the curve (AUC) values for one-, three- and five-years of survival were 0.734, 0.718 and 0.707, respectively, then we validated the prognostic model in International Cancer Genome Consortium database. Gene set enrichment analysis displayed that these four prognostic genes were primary enriched pathways related to cell cycle regulation. Our finding demonstrated that circELMOD3 could affect the relative expression levels of N-cadherin, E-cadherin, CDK4, CDK6 and CyclinD1 proteins.

**Conclusion:**

we constructed a novel ceRNA network based on circELMOD3, to comprehensively characterizing the prognosis of HCC, providing valuable insights for the therapy and prognosis of HCC.

## Introduction

Liver cancer is a major global health concern, with approximately 906,000 new cases and 830,000 deaths reported worldwide in 2020. Among malignant tumors, liver cancer ranks as the second most common cause of death in males. HCC is the most common histological subtype of liver cancer, accounting for more than 90% of cases (Sung, et al., 2021). In China, HCC ranks second in terms of fatality and fourth in terms of incidence among malignant tumors, posing significant public health hazards (Zheng, et al., 2019). Currently, available treatments for HCC encompass liver transplantation, hepatectomy, ablative therapy, radiation therapy, transcatheter arterial chemoembolization, and systemic anti-cancer treatment (Ikeda, et al., 2018). However, due to the high malignancy of HCC and the limitations of hepatectomy, patient prognosis remains poor (Forner, et al., 2018). The use of chemotherapy drugs is frequently limited by drug resistance, resulting in serious systemic toxic reactions (Biswal and Mehta, 2018). Despite advances in liver cancer treatment techniques, the 5-year survival rate for advanced HCC patients remains below 15%, primarily due to late-stage detection and high rates of surgical recurrence and metastasis (Zeng, et al., 2018). It is crucial to improve the survival rate of HCC patients, thus the creation of reliable prognostic models for discovering new therapeutic targets remains essential.

Circular RNAs (circRNAs) are non-coding RNAs produced through reverse splicing of precursor mRNA, comprising exons, introns, or both (Kristensen, et al., 2019). These circular RNA molecules lack 3′polyadenylated tails and 5′caps. Conferring resistance to exonuclease degradation within cells (Yang, et al., 2022), consequently, circRNAs exhibit prolonged half-life compared to traditional linear RNA molecules (Ashwal-Fluss, et al., 2014; Rybak-Wolf, et al., 2015), rendering them promising candidates for stable molecular markers. The concept of competitive endogenous RNA (ceRNA) was initially introduced in 2011 (Salmena, et al., 2011), Certain non-coding RNAs (ncRNAs), such as circRNAs, contain miRNA response elements that competitively bind miRNAs with mRNAs, thereby indirectly controlling mRNA expression and participating in the complex network of post-transcriptional regulation (Karreth and Pandolfi, 2013), Numerous studies have illustrated the crucial function of the ceRNA regulatory network in various diseases (Wang, et al., 2022; Kristensen, et al., 2022; Li, et al., 2020), suggesting its potential as a promising therapeutic target and a highly effective molecular marker.

This study is based on our previous findings that circELMOD3 expression is significantly downregulated in HCC tissues compared with controls, and it has the potential to be a highly effective diagnostic marker for liquid biopsy in HCC patients, as well as to inhibit HCC progression through miR-6864-5p/TRIM13/p53 signaling pathway (Lai, et al., 2023), The rapid development of bioinformatics allows its wide application in life science research. For this research, we applied various bioinformatic analyses to construct a ceRNA network based on circELMOD3. Subsequently, we identified prognostic genes and constructed a prognostic risk model. GSEA was employed to analyze the biological pathways of prognostic genes. Finally, through experimental validation, we confirmed the regulates of circELMOD3 on both the cell cycle and the molecules involved in the epithelial-mesenchymal transition (EMT) process. In conclusion, our research points to the possibility that circELMOD3 and its associated ceRNA network might be valuable indicators for the prognosis of HCC and targets for therapeutic intervention.

## Materials and methods

### Data acquisition and processing

The TCGA-LIHC database (https://portal.gdc.cancer.gov/) was used to download the associated clinical data along with the mRNA expression profiling data (including 374 HCC and 50 paracancer tissues samples) and miRNA expression profiling data (including 375 HCC and 50 paracancer tissue samples). The gene expression profiling data of the LIRI-JP (Liver cancer-RIKEN, Japan) cohort, comprising 232 tumor samples, was acquired from the ICGC database (https://dcc.icgc.org/) together with its corresponding clinical information. Demographic and clinicopathological characteristics of HCC patients in the TCGA-LIHC and LIRI-JP cohorts are summarized in Supplementary Table S1. Additionally, gene expression profiles for the GSE54236 and GSE101685 datasets were retrieved from the GEO database.

### The analysis of differentially of miRNA and mRNA

Firstly, genes with expression level of 0 in more than half of the samples were excluded. The differential expression analysis of mRNA and miRNA between HCC and paracancer tissues was then calculated using DESeq2 R package. The following criteria were used to identify differential genes: adjusted *p* < 0.05 and |log2 (fold change)| >1.

### Construction of the circELMOD3-miRNA-mRNA network

The downstream target miRNAs of circELMOD3 were initially predicted using the circMine database (http://www.biomedical-web.com/circmine/home) with default parameters. Subsequently, the predicted miRNAs were intersected with upregulated miRNAs identified in the TCGA-LIHC dataset. The downstream target mRNAs of these miRNAs were predicted using TargetScan 8.0 (https://www.targetscan.org/vert_80/) with default parameters and miRWalk 2.0 databases (http://mirwalk.umm.uni-heidelberg.de/), with binding sites restricted to the 3′UTR region. Only mRNAs predicted in both databases were retained. These selected mRNAs overlapped with downregulated mRNAs in TCGA-LIHC. Finally, the circELMOD3-miRNA and miRNA-mRNA interactions were used to construct the ceRNA network via Cytoscape v3.9.1 software.

### Functional enrichment analysis

To evaluate the potential biological functions and pathways of mRNAs in the ceRNA network, we performed Kyoto Encyclopedia of Genes and Genomes (KEGG) and Gene Ontology (GO) enrichment studies using the “clusterProfiler” package. *p*-value less than 0.05 was defined as cutoff criterion.

### Survival analysis

We initially collected clinical data, excluded samples with missing survival time or event status, and removed patients with an overall survival time of less than 1 month prior to performing survival analysis. The Kaplan-Meier curve was employed to perform survival analysis on miRNA and mRNA through the utilization of the “survival” and “survminer” packages. *p*-value less than 0.05 was defined as cutoff criterion.

### CeRNA network-related prognostic model

LASSO is a penalized regression technique that can be employed to screen variables from high-dimensional data for constructing prognostic models. Firstly, prognostic genes in the ceRNA network were identified using univariate Cox regression, with *p* < 0.05 being defined as cutoff criterion. Subsequently, the relevant genes that influence prognosis were identified via the glmnet LASSO regression method. The following formula is utilized to calculate the risk score: risk score = (EXP_gene1_ × coefficient_gene1_) +(EXP_gene2_ × coefficient_gene2_) + (EXP_genex_ × coefficient_genex_). Patients were categorized into high-risk and low-risk groups according to their median risk score, and the survival difference between the two groups was then assessed using a log-rank test. Furthermore, model performance was evaluated by employing the ‘timeROC’ package to draw the receiver operating characteristic (ROC) curve. We validated the prognostic risk model from the LIRI-JP cohort by calculating individual patient risk scores according to the aforementioned formula. Furthermore, we evaluated predictive value using time-dependent ROC curve analysis and Kaplan-Meier analysis.

### Construction and evaluation of the nomograms

The nomogram is widely used for prognostic prediction in tumor patients, univariate and multivariate Cox regression were performed to identify independent prognostic predictors in HCC patients, including gender, stage, age and risk score. A nomogram incorporating these prognostic predictors was constructed using the “rms” package. The fitting and prediction power of our prognostic model were evaluated by plotting calibration curves.

### Gene set enrichment analysis

The TCGA-LIHC dataset was subjected to GSEA. Based on the median gene expression levels, HCC patients were stratified into high- and low-expression groups to conduct GSEA. Pathways exhibiting significant enrichment in these two groups were subsequently analyzed. The criteria for statistical significance were FDR < 0.25 and *p* < 0.05.

### Human samples, RNA extraction and real-time quantitative RT-PCR (qRT-PCR)

HCC and adjacent tissues were collected as previously described (Lai, et al., 2023). The informed consent was obtained from each patient and the study was approved by the Ethics Committee of Guangxi Medical University (20220146). Human liver cancer cell line MHCC97H was purchased in CellCook Biotech Co., Ltd. and Hep3B cell line from Cell bank of the Chinese academy of sciences. The cell culture and cell transfection procedure were performed following earlier depicted protocols (Lai, et al., 2023). Trizol reagent was used to extract total RNA, and the absorbance was measured by a microplate reader. Using PrimeScript RT Master Mix (Takara, RR036A), total RNA was reverse-transcribed into cDNA, followed by qRT-PCR analysis on a Stepone Plus system (Applied Biosystem, United States) with a qPCR detection kit (GenStar, A303). GAPDH served as the internal reference for mRNA quantification. We used the 2^-△△CT^ method to perform a relative gene expression study. Supplementary Table S2 lists the primer sequences that were used in this research.

### Western blot analysis

The Western blotting analysis was performed following earlier depicted protocols (Lai, et al., 2023). The primary antibodies used were N-cadherin (1:1000, 22018-1-AP), E-cadherin (1:2500, 20874-1-AP), CDK4 (1:2000, 11026-1-AP), CDK6 (1:2500, 14052-1-AP), CyclinD1 (1:10000, 60186-1-lg), GAPDH (1:10000, 60004-1-lg), Tubulin (1:6000, 11224-1-AP) and all antibodies are sourced Proteintech. Subsequently, the membrane was incubated with the secondary antibody for 1 hour at room temperature on a shaker. Protein bands were visualized by adding an enhanced chemiluminescence (ECL) substrate and imaging with an iBright FL1000 system.

### Statistical analysis

R software (version 4.3.2) and GraphPad Prism software (version 8.3) were used to perform data analyses. The relationship between two gene variables was investigated using Spearman correlation analysis, and the difference between the two groups was assessed using the Wilcoxon rank-sum test. Univariate Cox analysis was applied to identify potential risk factors affecting prognosis. Using multivariate Cox analysis, independent prognostic factors were identified. The predictive accuracy of the HCC prognostic model was validated through receiver operating characteristic (ROC) curve analysis. All *in vitro* experiments included three independent biological replicates, and data are presented as mean ± standard deviation. *p*-value less than 0.05 was defined as cutoff criterion.

## Results

### Identification of differentially expressed miRNAs and mRNAs in the TCGA-LIHC database

**FIGURE 1:**
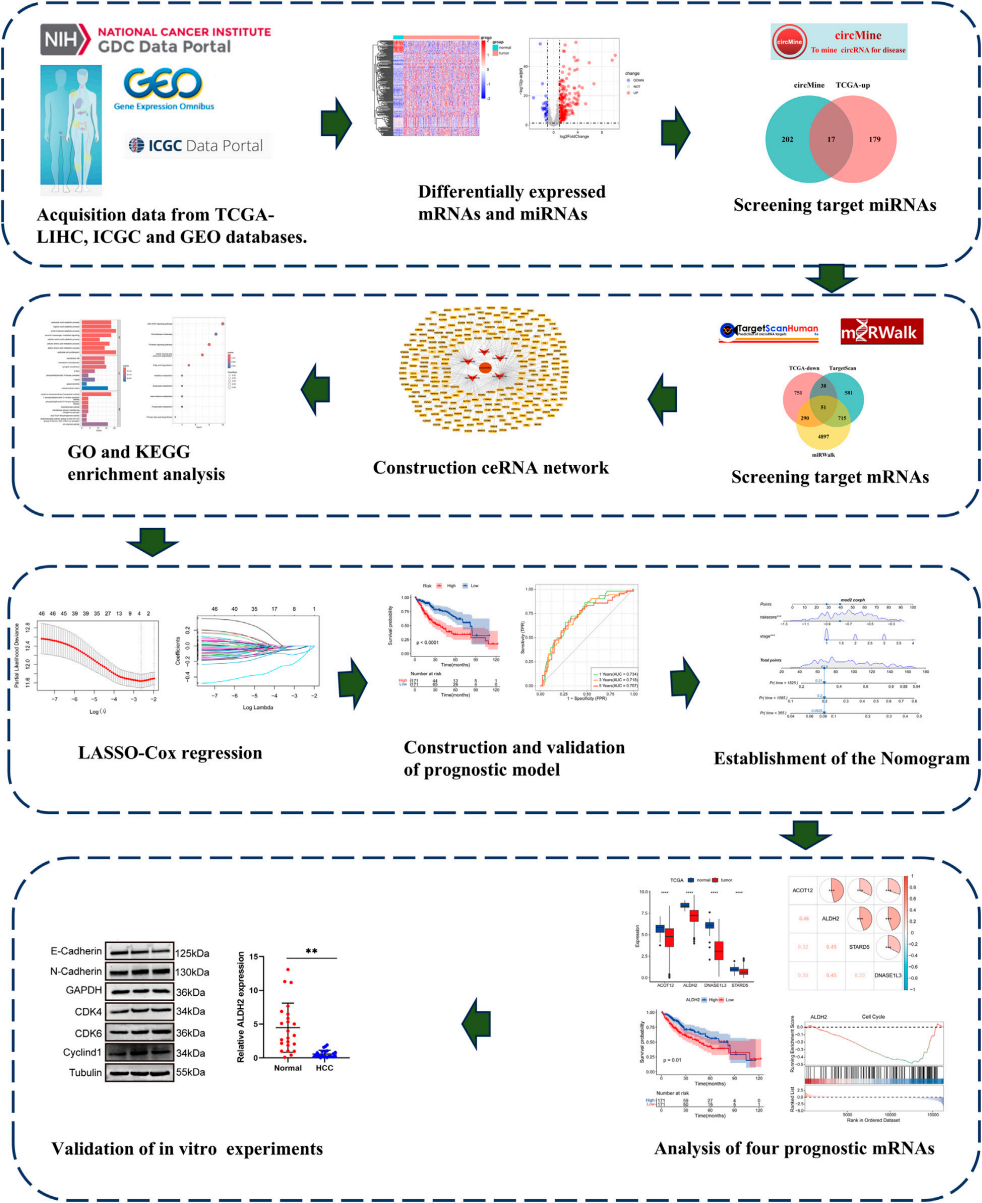
The Flow diagram of this study design.

The detailed flow chart of study process is shown in Figure 1. Differentially expressed mRNAs and miRNAs in HCC were performed using the TCGA-LIHC dataset. The results showed a total of 3982 significantly dysregulated mRNAs, with 2853 showing upregulated and 1130 displaying downregulated (Supplementary Figures S1A, B). Furthermore, 225 differentially expressed miRNAs were found, of which 196 were upregulated and 29 were downregulated (Supplementary Figures S1C, D).

### Prediction and analysis of binding miRNAs of circELMOD3

**FIGURE 2:**
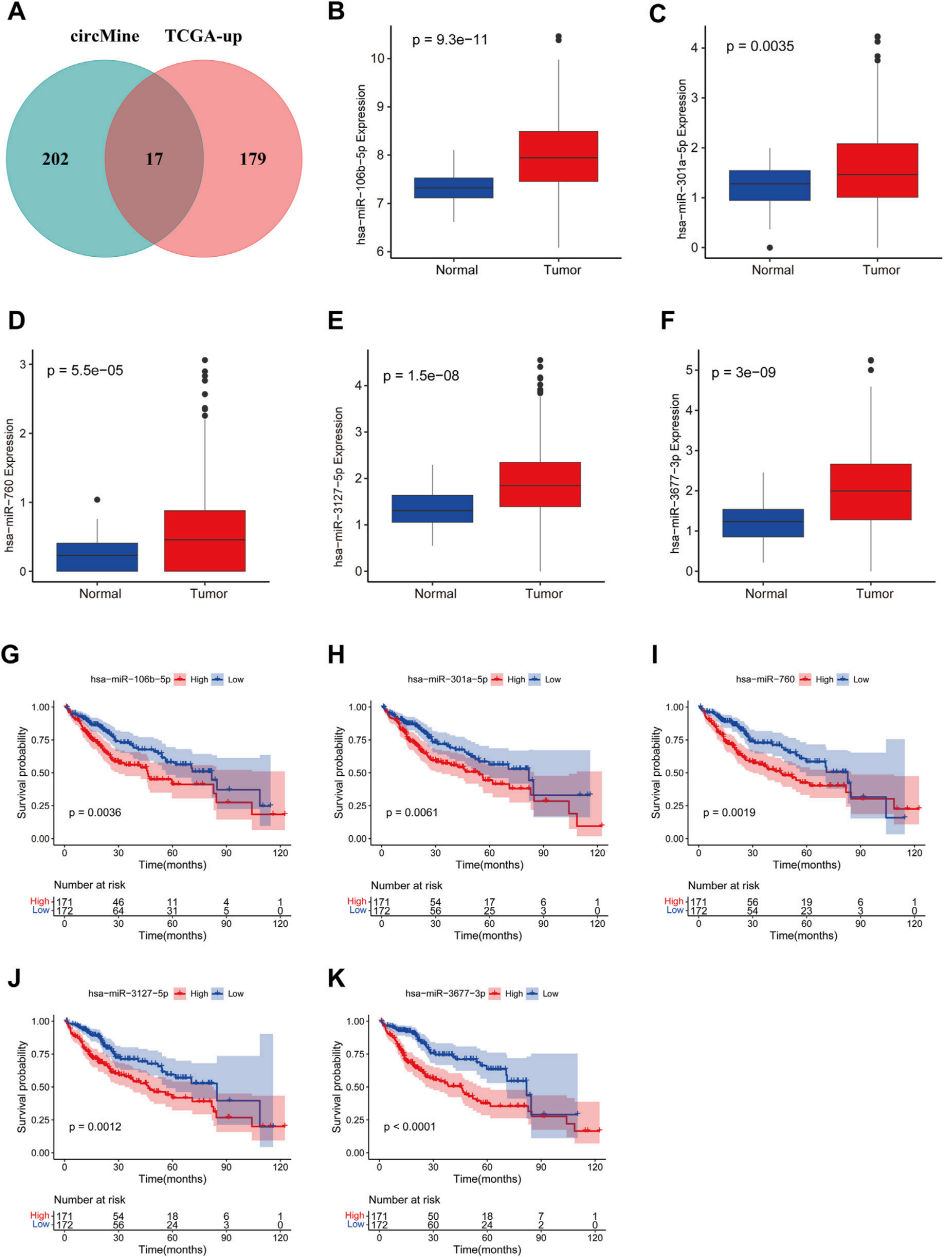
Identification and analysis of miRNAs binding to circELMOD3 in TCGA-LIHC. **(A)** Veen diagram showing the target miRNA of circELMOD3 in the circMine database and upregulated miRNA in TCGA-LIHC. The expression levels of **(B)** miR-106b-5p, **(C)** miR-301a-5p, **(D)** miR-760, **(E)** miR-3127-5p and **(F)** miR-3677-3p were evaluated in TCGA-LIHC. Kaplan-Meier survival analysis of **(G)** miR-106b-5p, **(H)** miR-301a-5p, **(I)** miR-760, **(J)** miR-3127-5p and **(K)** miR-3677-3p in TCGA-LIHC, cut-off: median of miRNA expression.

To identify potential miRNAs that might bind to circELMOD3, the circMine database was utilized. The ceRNA hypothesis suggests that the expression of miRNA and circRNA should be negatively related. By intersecting upregulated miRNAs from the TCGA-LIHC dataset with circMine-predicted miRNAs, we identified 17 candidate miRNAs (Figure 2A). Considering circELMOD3 was previously found to affect various biological functions of HCC cells, we further explored the relationship between circELMOD3-binding miRNAs and the survival of HCC patients. Log-rank tests and univariate Cox regression analysis were carried out on these miRNAs (Supplementary Tables S3, 4). Prognostic miRNAs were selected based on significance thresholds of *p* < 0.05 in both analyses, yielding five miRNAs (including hsa-miR-106b-5p, hsa-miR-301a-5p, hsa-miR-760, hsa-miR-3127-5p, hsa-miR-3677-3p) with potential circELMOD3 interactions. The relative expression levels of five prognosis-related miRNAs in HCC were significantly upregulated compared to paracancer tissues in the TCGA-LIHC database (Figures 2B–F). Kaplan-Meier analysis displayed that high expression levels of five identified miRNAs were associated with poor overall survival in HCC patients (Figures 2G–K), The findings suggest that these five miRNAs may have oncogenic properties in HCC.

### Construction the ceRNA network

**FIGURE 3:**
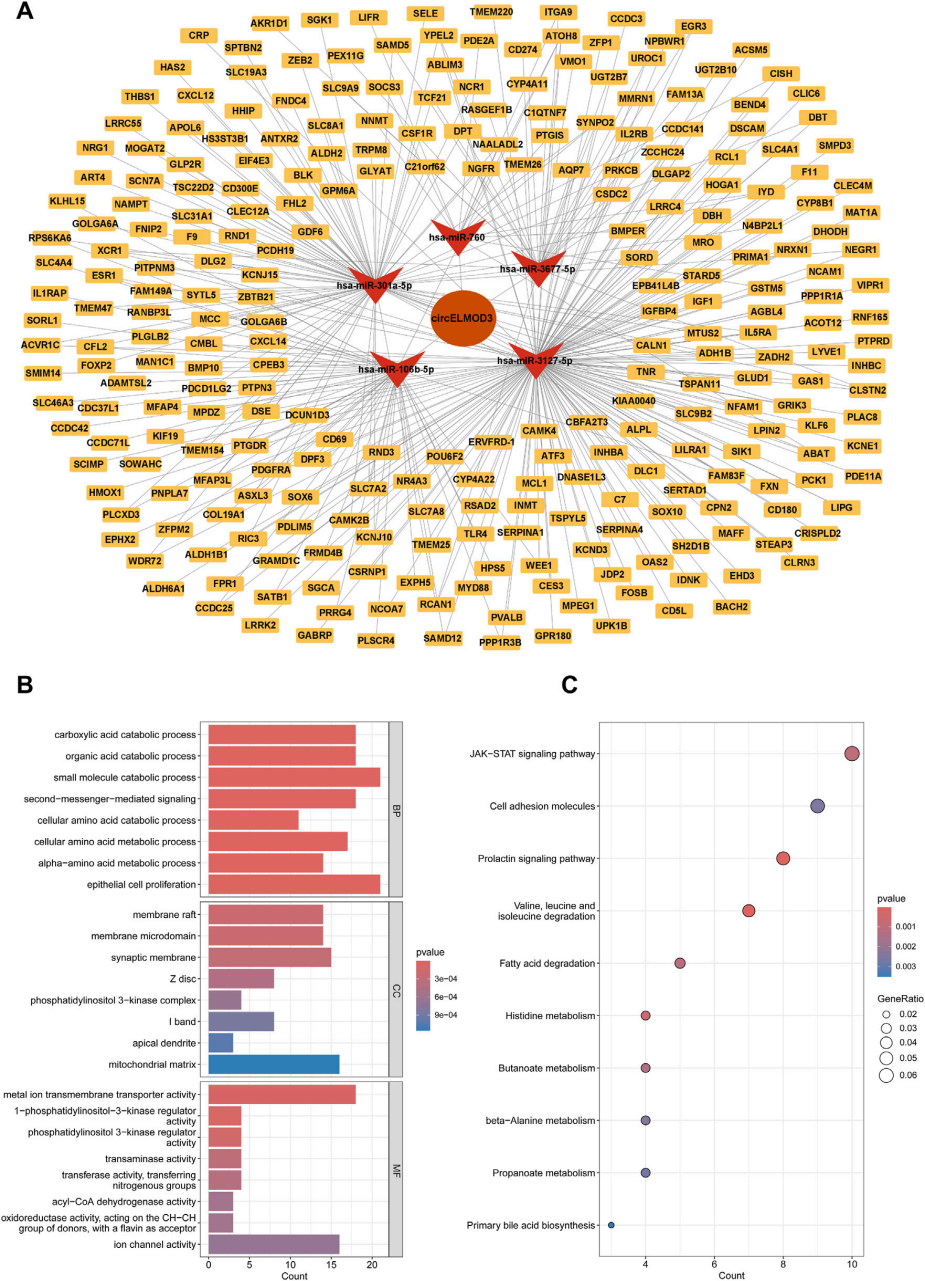
Construction of a circELMOD3-associated ceRNA network. **(A)** The circELMOD3-miRNA-mRNA network was built based on predicted relationship. **(B)** Enrichment analysis revealed the top 8 Gene Ontology term **(C)** and the top 10 KEGG pathways were identified in this ceRNA network. BP: Biological Process; CC: Cellular Component; MF: Molecular Function.

To comprehensively comprehend the role of circELMOD3 through ceRNA network in HCC, we constructed a circELMOD3-centered miRNA-mRNA interaction network. Candidate mRNAs potentially binding to the five miRNAs were predicted using miRWalk and TargetScan databases. Subsequently these predicted mRNAs were further intersected with downregulated mRNA identified in the TCGA-LIHC database, resulting in a set of candidate mRNAs (Supplementary Figures S2A–E). Finally, by integrating the predictive relationships between circELMOD3-miRNA and miRNA-mRNA interactions, we constructed and visualized a ceRNA regulatory network comprising circELMOD3, 5 miRNAs, and 274 mRNAs using Cytoscape software (Figure 3A).

### Functional enrichment analysis

To explore the regulatory role of circELMOD3 in ceRNA network, we carried out functional enrichment studies using KEGG and GO. The results of GO analysis exhibited that biological functions (BP) primarily encompassed organic acid catabolic process, carboxylic acid catabolic process, and epithelial cell proliferation. In terms of cellular component (CC), it was mainly associated with membrane raft, membrane microdomain, and phosphatidylinositol 3-kinase complex. Regarding molecular function (MF), the circELMOD3 regulatory gene was predominantly linked to metal ion transmembrane transporter activity, phosphatidylinositol 3-kinase regulator activity, and transaminase activity (Figure 3B). Furthermore, the KEGG pathway enrichment analysis demonstrated that circELMOD3 regulatory genes are mainly involved in cell adhesion molecules, JAK-STAT signaling pathway, as well as cell adhesion molecules (Figure 3C). These findings suggest a pivotal role for circELMOD3 in HCC development.

### Construction of ceRNA network related prognostic model in TCGA-LIHC

**FIGURE 4:**
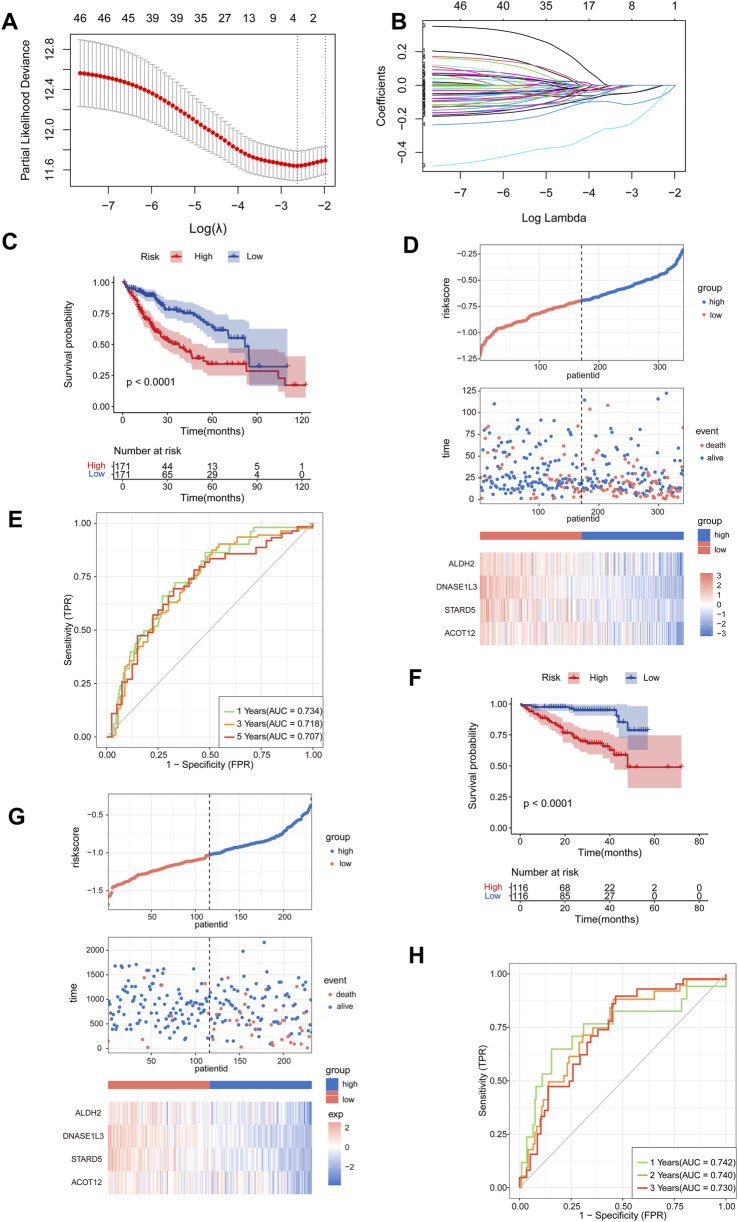
Construction of a prognostic model related to the ceRNA network. **(A, B)** The number of factors was determined using LASSO analysis and the LASSO coefficient profiles of 46 prognostic mRNAs were obtained. **(C)** Survival analysis was conducted for the high- and low-risk groups using Kaplan-Meier curves in TCGA-LIHC, cut-off: median of riskscore. **(D)** The risk score distributions, patients’ survival status, and the expression heat map of four identified genes were compared between the high- and low-risk groups in TCGA-LIHC. **(E)** Time-ROC curve was produced to predict OS at one-, three- and five-years in TCGA-LIHC. **(F)** Survival analysis was performed to assess the survival of patients in the high- and low-risk groups using Kaplan-Meier curves within the LIRI-JP cohort, cut-off: median of riskscore. **(G)** The risk score distributions, patients’ survival status, and the expression heat map of four identified genes were compared between the high- and low-risk groups in LIRI-JP cohort. **(H)** Time-ROC curve for OS prediction at one-, two- and three-years in LIRI-JP cohort.

From the TCGA-LIHC cohort, 342 HCC patients with complete survival data and a minimum survival time of 1 month were included based on predefined screening criteria. We employed univariate Cox regression analysis with *p* < 0.05 and HR < 1 as the thresholds to screen for prognostic related genes in the circELMOD3-regulated ceRNA network, yielding 46 candidate genes (Supplementary Tables S5, 6). Subsequently, we further employed LASSO regression analysis to screen genes, selected the optimal penalty coefficient λ (λ = 0.072) by 10-fold cross-validation, and obtained 4 genes with non-zero coefficients (*ALDH2, DNASE1L3, STARD5, ACOT12*), and the following formula was used to determine the risk score: risk score = (EXP_ALDH2_*-0.02341441) + (EXP_DNASE1L3_*-0.09186373) + (EXP_STARD5_*-0.15196182) + (EXP_ACOT12_*-0.02724502) (Figures 4A, B). HCC Patients were categorized as low-risk or high-risk depending on their median risk scores. Patients in the low-risk group exhibit higher survival probabilities compared to those in the high-risk group (Figure 4C). Furthermore, expression levels of all four prognostic genes were considerably lower in the high-risk group than in the low-risk group; patient mortality increased significantly with increasing risk values (Figure 4D). The time-dependent receiver operating characteristic (time-ROC) curve was used to assess the predictive power of this model; the area under the 1-year, 3-year, and 5-years ROC curves, respectively, were 0.734, 0.718, and 0.707 (Figure 4E).

### Validation of ceRNA network related prognostic model in LIRI-JP

To validate the robustness of the model, patients in the LIRI-JP cohort were stratified into low- and high-risk groups based on the same risk score calculation method used for the TCGA-LIHC cohort. The results showed that compared to those with higher risk scores patients, with lower risk scores patients exhibited higher survival rates and decreased mortality (Figure 4F). Furthermore, prognostic genes were considerably downregulated in the high-risk group compared to the low-risk group, patient mortality increased significantly with increasing risk values (Figure 4G). Additionally, time-ROC curve analysis displayed strong predictive ability for the LIRI-JP cohort, with AUC values of 0.742, 0.740, and 0.730 for predicting one-, two-, and three-year survival respectively (Figure 4H).

To assess prognostic stability of the model across diverse clinical subgroups, we performed stratified analyses based on age (≥65 years vs. < 65 years), sex (female vs. male), and stage (III-IV vs. I-II). The findings consistently showed a significantly lower risk of death in the low-risk group compared with the high-risk group. Although the survival difference in the female subgroup was not statistically significant, the low-risk group still showed a lower mortality risk when follow-up duration was limited to less than 70 months. (Supplementary Figures S3A–C). Similarly consistent outcomes were observed when performing stratified analyses within LIRI-JP cohort (Supplementary Figures S3D–F).

### Establishment of the nomogram

**FIGURE 5:**
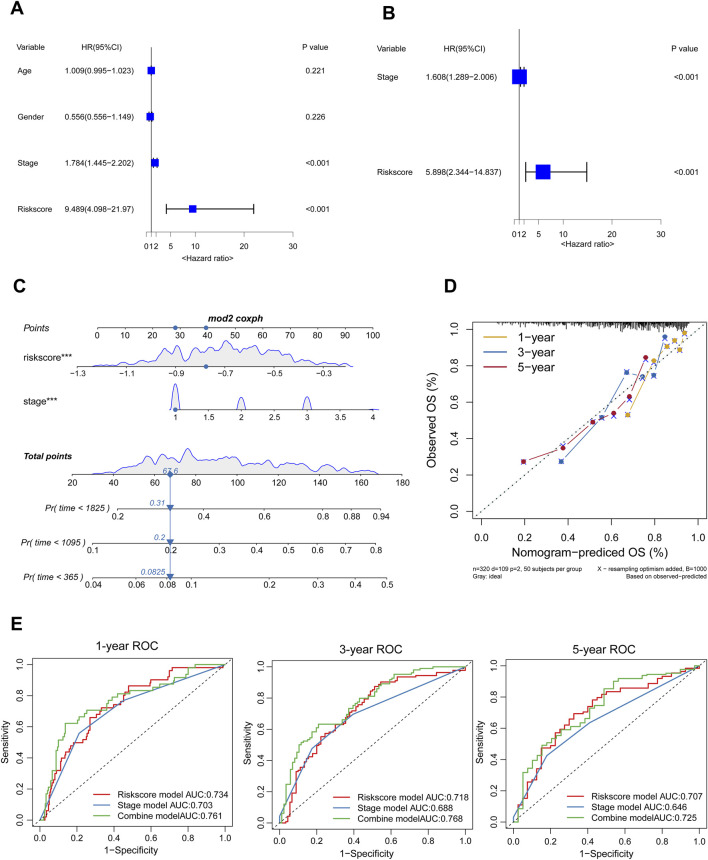
Establishment of the Nomogram. **(A, B)** The forest plot displayed results of univariable and multivariable cox analysis in the TCGA-LIHC. **(C)** A nomogram was developed to predict the one-, three-, five-years survival rates of HCC patients **(D)** The calibration chart was employed to evaluate the degree of coherence between the observed OS and the expected OS. **(E)** Time-ROC curve analysis of the combined model, risk score model, and stage model.

We developed a nomogram integrating the risk score (calculated as the sum of the expression levels of four LASSO-selected genes multiplied by their respective coefficients) and stage to enhance the clinical utility of the prognostic model in the TCGA-LIHC cohort. Firstly, the study employed univariate Cox regression analysis to investigate the potential prognostic predictive value of the risk score and specific clinical factors. The results demonstrated significant association between both the risk score (HR = 9.489, 95%CI: 4.098–21.970) and stage (HR = 1.784, 95%CI: 1.445–2.202) with overall survival in HCC patients (Figure 5A). Furthermore, multivariate cox regression analysis demonstrated that both the risk score (HR = 5.898, 95%CI: 2.344–14.837) and stage (HR = 1.608, 95%CI: 1.289–2.006) independently influenced OS among HCC patients (Figure 5B). Finally, we constructed nomograms incorporating risk scores and stage to accurately predict one-, three-, and five-year survival for HCC patients (Figure 5C). Higher total points on the nomogram correlated with poorer survival outcomes; moreover, the calibration plots for one-, three-, and five-year survival probabilities showed great concordance with actual observations (Figure 5D). Moreover, time-ROC analysis shown that, in comparison to the risk score and stage, the combined nomogram performed best in predicting the overall survival of HCC patients (AUC = 0.761 at 1 year, 0.768 at 3 years, and 0.725 at 5 years) (Figure 5E).

### Key gene expression, correlation, prognosis and GSEA analysis

**FIGURE 6:**
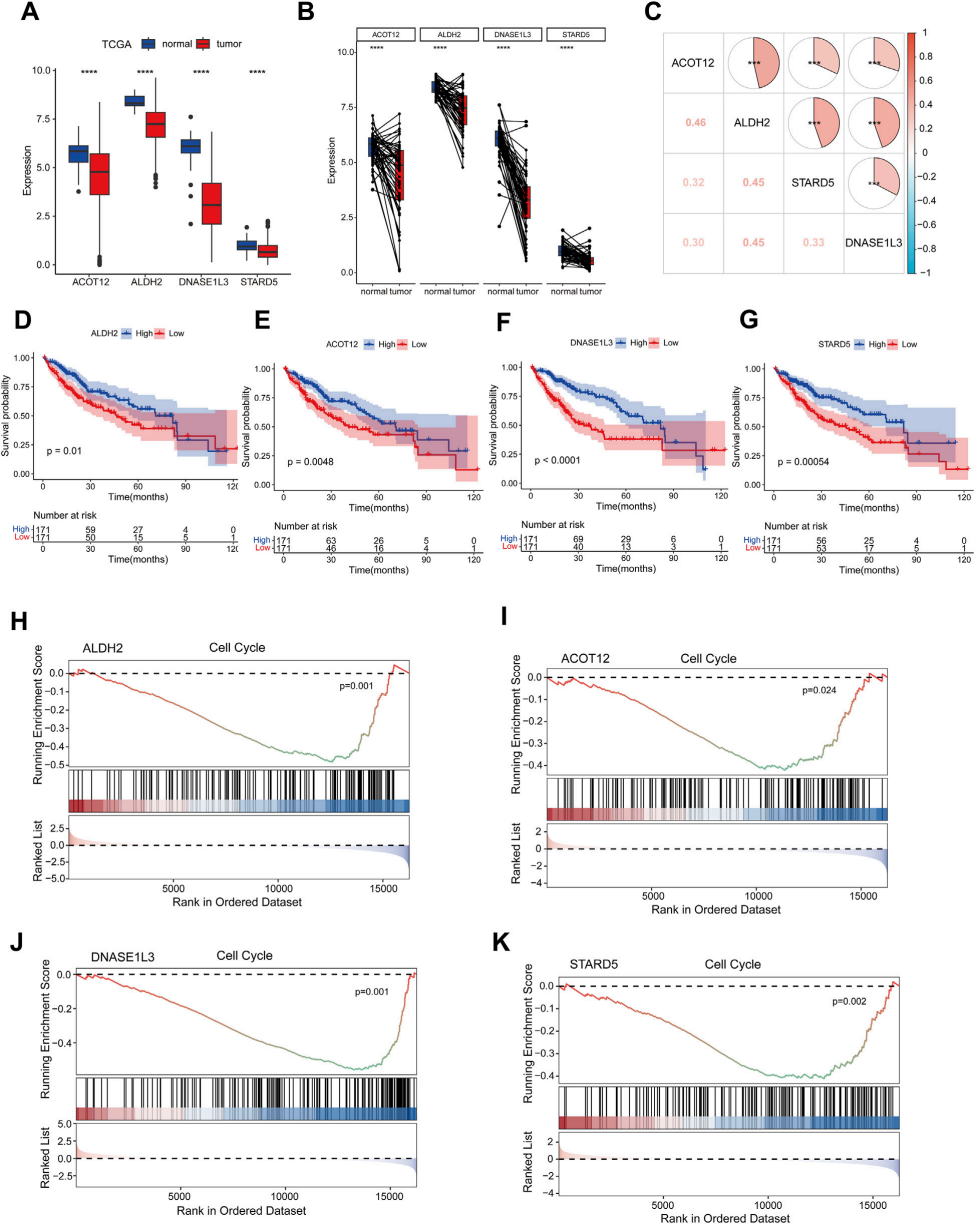
Key gene expression, correlation, prognosis and GSEA analysis. **(A)** The expression levels of four prognostic genes were analyzed in TCGA-LIHC dataset. **(B)** Paired sample expression analysis for the four prognostic genes in TCGA-LIHC. **(C)** The correlation analysis of four prognosis-related genes using spearman analyses. **(D–G)** Kaplan-Meier survival analysis of four prognosis genes, cut-off: median of mRNA expression. **(H–K)** Results of GSEA enrichment analysis of key genes. ns indicated no significance, *p < 0.05, **p < 0.01, ***p < 0.001, ****p < 0.0001.

To further characterize the four prognostic genes, we observed their downregulation in HCC using data from the TCGA-LIHC database (Figure 6A), as well as in paired samples (Figure 6B). These findings were validated in two independent GEO datasets (Supplementary Figures S4A, B). Correlation analysis revealed a positive association among the four prognostic genes (Figure 6C). Additionally, Kaplan-Meier survival analysis demonstrated that high expression of *ALDH2*, *DNASE1L3*, *STARD5*, and *ACOT12* was associated with prolonged survival in HCC patients (Figures 6D–G). GSEA results showed that all four prognostic genes were negatively correlated with the cell cycle pathway, indicating that relatively high expression levels of the four prognostic genes could inhibit the cell cycle pathway compared with low expression levels. (Figures 6H–K).

### circELMOD3 may be involved in cell cycle, EMT-related signaling pathway

**FIGURE 7:**
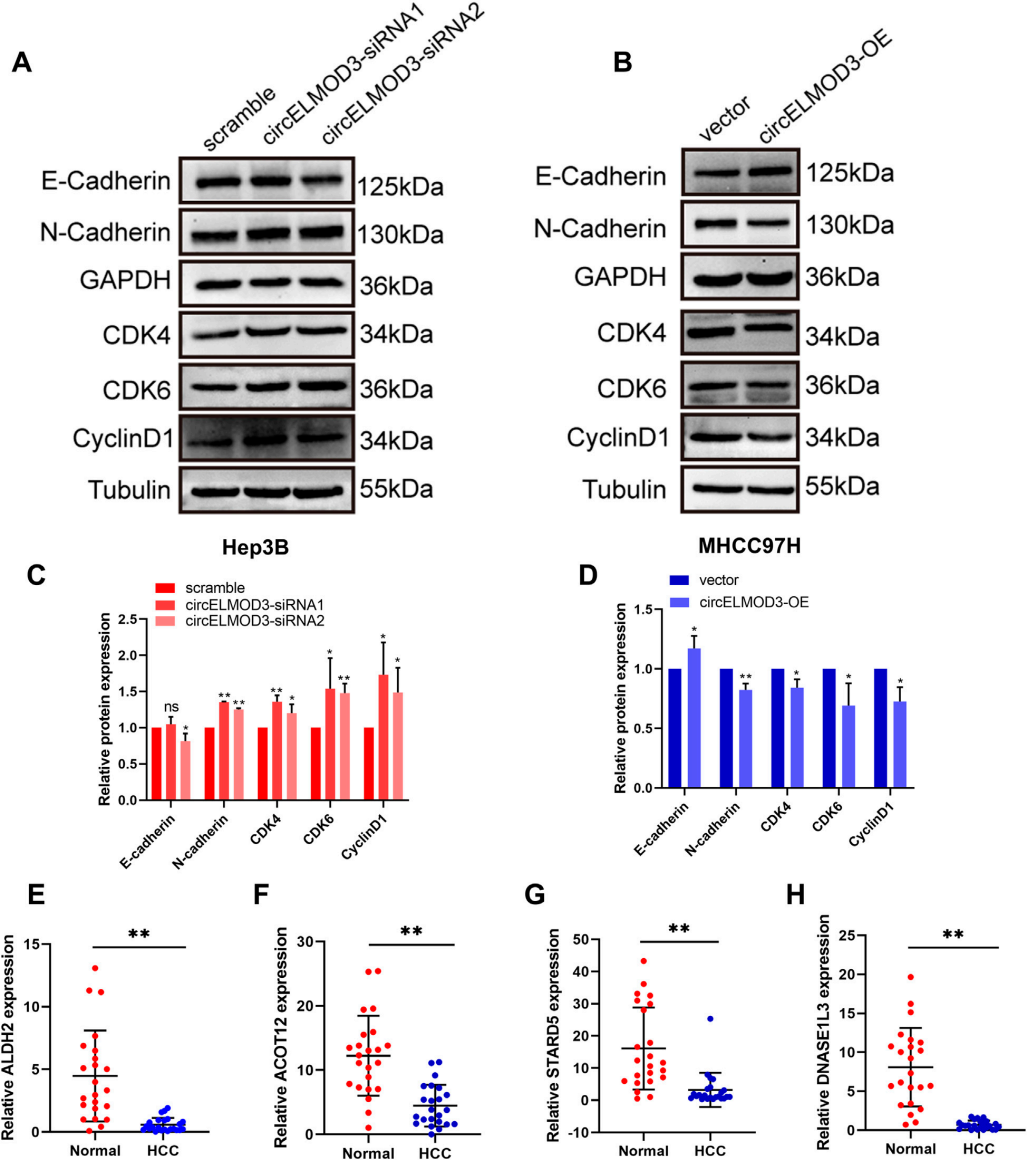
circELMOD3 regulated HCC progression through multiple pathways. **(A–D)** Western blot analysis was used to detect the protein levels of E-cadherin, N-cadherin, CDK4, CDK6, CyclinD1 when circELMOD3 was silenced in Hep3B cells or overexpressed in MHCC97H cells. **(E–H)** Relative expression levels of *ALDH2*, *DNASE1L3*, *STARD5*, *ACOT12* mRNA in 23 pairs of HCC and paracancer tissue samples. GAPDH was used as an internal control for N-cadherin and E-cadherin. Tubulin was used as an internal control for CDK4, CDK6, and CyclinD1; n = 3; ns indicated no significance, *p < 0.05, **p < 0.01.

Based on the GSEA results indicating the association of the four prognostic genes with the cell cycle pathway, and KEGG enrichment analysis showed that they were related to cell adhesion molecule pathway. We hypothesized that circELMOD3 might be related to cell cycle and EMT pathways. Subsequently, Western blot was performed to verify the expression levels of cell cycle pathway-related proteins CDK4, CDK6, CyclinD1 and EMT-related markers N-cadherin and E-cadherin. The results exhibited that silencing circELMOD3 in Hep3B cells led to an increase in the relative expression levels of CDK4, CDK6, CyclinD1 and N-cadherin proteins while decreasing the relative expression levels of E-cadherin proteins (Figures 7A, C). Conversely, overexpression of circELMOD3 in MHCC97H cells resulted in a decreased in the relative expression levels of CDK4, CDK6, CyclinD1 and N-Cadherin proteins with an increase observed for E-cadherin protein (Figures 7B, D). To further validate the expression levels of the four prognostic related genes in HCC tissues, we performed qPCR validation using 23 pairs of HCC tissues. The results showed that *ALDH2*, *ACOT12*, *STARD5* and *DNASE1L3* were significantly downregulated in HCC tissues compared with paracancer tissues. (Figures 7E–H).

## Discussion

With its high rate of postoperative recurrence and complex biology, HCC is the most frequent cause of cancer-related mortality worldwide. Because of its stability and specificity, circRNA can be used as a therapeutic target and prognostic marker in tumor progression. In this study, our prognostic model based on the circELMOD3-miRNA-mRNA network had a good prediction of the prognosis of HCC patients. Besides, we also found that circELMOD3 may be involved in the progression of HCC through the cell cycle and EMT processes. Our research indicated that circELMOD3 may be a potential therapeutic target as well as a prognostic marker.

Based on previous studies, we plan to further investigate the function of circELMOD3 in the prognosis of HCC patients. Accumulating evidence highlights the critical involvement of miRNAs in regulating physiological and pathological processes, particularly in malignancies such as HCC. It has been demonstrated that circRNAs possess abundant miRNA binding sites, suggesting their potential as sponges for adsorbing corresponding miRNAs and thereby regulating the expression of target genes through ceRNAs. Using the circMine database and integrating upregulated miRNAs from the TCGA-LIHC dataset, we identified 5 candidate miRNAs targeting circELMOD3: hsa-miR-106b-5p, hsa-miR-301a-5p, hsa-miR-760, hsa-miR-3127-5p, hsa-miR-3677-3p. Survival analysis revealed that high expression of these miRNAs correlated with poorer overall survival in HCC patients. Previous studies have shown miR-106b-5p could serve as a prognostic marker for HCC patients, and play a carcinogenic role by regulating the target gene *RUNX3* (Gu, et al., 2019). Furthermore, research showed that miR-3677-3p binds to *FBXO31* and inhibit its expression in hepatitis B-associated hepatocellular carcinoma, this inhibition causes FOXM1 to be less ubiquitinated and degraded, which in turn encourages the growth of HCC and sorafenib resistance (He et al., 2023a).

Subsequently, we used TargetScan and miRWalk databases, combined with downregulated mRNAs in HCC from the TCGA-LIHC database, to predict target genes of the five candidate miRNAs. To visualize the regulatory interactions among circELMOD3, 5 miRNAs, and 274 mRNAs, we constructed a ceRNA network using Cytoscape. Furthermore, by performing GO and KEGG enrichment analysis, it was discovered that these 274 mRNAs are involved in multiple cancer-related biological processes. For example, JAK/STAT pathway is an important cellular cascade that plays an important role in the control of cell proliferation, invasion and apoptosis, as well as the regulation of immune and inflammatory responses. This signaling pathway also plays a crucial role in various stages of tumorigenesis, including epithelial-mesenchymal transition (EMT) and metastasis (Park, et al., 2023). SRRM1 is highly expressed in liver cancer tissues. *SRRM1,* which is overexpressed in HCC tissues, promotes tumor progression by activating the JAK/STAT pathway. Knockdown of *SRRM1* suppresses proliferation, induces apoptosis, and reduces invasion by inhibiting this pathway (Song and Ma, 2022). The intracellular amino acid metabolism pathway is crucial for HCC development. Multi-omics studies demonstrate that Gamabufotalin exerts anti-HCC effects through STAMBPL1-mediated regulation of amino acid metabolism (Zheng, et al., 2024). Similarly, Catharanthine inhibit the growth and proliferation of liver cancer cells by regulating amino acid metabolism and cholesterol metabolism, thereby suppressing tumorigenesis (Feng, et al., 2021).

We applied Cox and LASSO regression analyses to the ceRNA network to construct a prognostic model for HCC. Subsequently, we developeed a prognosis risk model consisting of four genes (*ALDH2*, *DNASE1L3*, *ACOT12*, *STARD5*). Patients with higher risk scores exhibited significantly shorter survival compared to those in the low-risk group. The prognosis model had better predictive survival probabilities for HCC patients. Furthermore, validation in the LIRI-JP cohort further confirmed its robust prognostic performance. One study reported (Liu, et al., 2023) a prognostic model constructed by a circRNA-associated ceRNA network containing seven hub genes, and another study (Zheng, et al., 2021), a prognostic prediction model for HCC patients based on pyroptosis-related genes, our model has higher AUC values at one, three, and five years compared to these models. Multivariate Cox analysis identified both the risk score and pathological stage as independent prognostic factors for HCC. Consequently, we constructed a nomogram incorporating both risk score and pathological stage to provide individualized prognosis predictions at 1-year, 3-year, and 5-year time points, the combined model greatly improved the prognostic prediction of HCC. Although Chen et al. reported a prognostic model based on five cuproptosis-related genes with robust performance in training and validation cohorts (Chen, et al., 2023), our model exhibited a higher 5-year AUC, which means that our risk score combined with stage model has good predictive power.

The stress-responsive protein STARD5 controls the equilibrium of intracellular and plasma membrane cholesterol (Létourneau, et al., 2012). *STARD5* methylation is closely associated with the prognosis of ccRCC patients (Qian, et al., 2020). A study showed that *STARD5* was downregulated in the TCGA-LIHC database, suggesting its potential as a biomarker for HCC prognosis and diagnosis. Additionally, it is implicated in cell cycle pathways and plays a crucial role in immune infiltration (Liu, et al., 2022). Acyl-CoA thioesterase 12 (*ACOT12*), a crucial molecule involved in cellular energy metabolism, glucose metabolism, as well as tumor development and progression. *ACOT12* expression was significantly downregulated in HCC tissues and decreased pancreatic coenzyme levels and facilitated HCC metastasis through epigenetic modulation of EMT (Lu, et al., 2019). Additionally, studies have demonstrated that *ACOT12* inhibits HCC by restricting glycerol ester biosynthesis via the Hippo signaling pathway (He et al., 2023b). Multiple bioinformatics studies have consistently confirmed the downregulation of *DNASE1L3* in various cancer (Chen, et al., 2021; Liu, et al., 2021; Ge, et al., 2023). Experimental investigations have further elucidated the tumor-suppressor role of *DNASE1L3* in HCC, *DNASE1L3* exerts its anti-HCC effects by impeding glycolysis (Xiao, et al., 2022) and impairing the advancement of the cell cycle by integrating with CDK2 (Sun, et al., 2022). Additionally, *DNASE1L3* impeded HCC progression by binding to beta-catenin thereby promoting its ubiquitination degradation and subsequently suppressing downstream signaling pathways involved in cell cycle and EMT (Li, et al., 2022). *ALDH2*, associated with glycolysis, exhibits prognostic value in HCC and breast cancer (Tang, et al., 2020). Functional experiment showed that *ALDH2* inhibits the invasion and migration of HCC cells while activating AMPK phosphorylation via a redox-dependent mechanism, thereby impeding the progression of HCC (Hou, et al., 2017). It also suppresses immune escape in HCC by disrupting the ROS/Nrf2 axis to induce autophagy (Hu, et al., 2022).

Intracellular biological processes and signaling pathways critically regulate tumor progression. The cell cycle is a crucial biological mechanism that controls cell division, growth, and genetic material replication. The abnormal regulation of cell cycle is the basis of abnormal proliferation of cancer cells (Malumbres and Barbacid, 2009). Among them, cyclin-dependent protein kinases (CDKs) are the core proteins that regulate the cell cycle process, so targeting cyclin is an effective method to prevent tumor growth. CDK4 and CDK6 inhibitors that target cell cycle mechanisms have already been introduced into the clinic and used to treat breast cancer, and may have a profound impact on targeted therapies for other tumor types (Álvarez-Fernández and Malumbres, 2020). Our study demonstrated that circELMOD3 modulates the expression of CDK4, CDK6, and cyclin D1, suggesting its role in suppressing HCC by impeding cell cycle progression. Cancer cell metastasis is a crucial factor for postoperative recurrence and poor prognosis of tumor patients. The biological process by which epithelial cells change into mesenchymal phenotypic cells is known as epithelial-mesenchymal transformation (EMT), and it is an early indicator of malignant tumor invasion and metastasis (Yang, et al., 2020). Previous studies have confirmed that circRNA plays a crucial role in regulating cancer cell metastasis and invasion through EMT process in HCC. For example, hsa_circ_0003288 can upregulates PD-L1 expression through sponge adsorption of miR-145, thereby activating PI3K/AKT pathway to promote EMT process and invasion of HCC cells (Xu, et al., 2021). In addition, we validated that circELMOD3 can affect the expression of N-cadherin and E-cadherin protein, which is essential for the EMT process. Collectively, these observations indicate that circELMOD3 potentially regulates HCC progression through diverse mechanisms involving ceRNA regulatory networks.

However, this study has several limitations. Firstly, additional experimental validation is required to confirm the accuracy of the ceRNA regulation network since it was mostly constructed using public databases and a number of bioinformatic analyses. Secondly, further research in a liver cancer cohort is necessary to confirm circELMOD3’s potential usefulness as a prognostic biomarker. Finally, the molecular mechanisms by which circELMOD3 regulates HCC progression particularly its associated signaling pathways require deeper exploration through *in vitro* and *in vivo* experiments and mechanistic studies.

## Conclusion

In summary, we constructed a circELMOD3-miRNA-mRNA network centered on circELMOD3 through comprehensive analysis of public databases and bioinformatics tools. Additionally, we identified four prognostic mRNAs (*ALDH2*, *DNASE1L3 ACOT12*, *STARD5*) in the ceRNA network. Based on these four genes, a prognostic model may have practical implications. Finally, experimental validation confirmed the impact of circELMOD3 on cell cycle and epithelial-mesenchymal transition signaling markers. These results suggest that circELMOD3 exerts multifaceted effects on HCC progression through intricate regulatory networks and may act as a valuable therapeutic target and prognostic marker for HCC.

## Data Availability

The original contributions presented in the study are included in the article/Supplementary Material, further inquiries can be directed to the corresponding author.
