# Supplementary material for: The regulatory role of the circELMOD3-associated ceRNA network in the progression and prognosis of hepatocellular carcinoma
Source: Front Genet. 2025 Apr 15;16:1521360. doi: 10.3389/fgene.2025.1521360 (PMC12037612; doi:10.3389/fgene.2025.1521360)
Supplement: Supplementary file 3 [file Table2.docx]

**Table S2**

**Table S1. Nucleic acid sequence information of the siRNA and PCR primer**

| siRNA | Sequence | |
| --- | --- | --- |
|  | sense | antisense |
| circELMOD3 siRNA1 | GGCUUUCAGGUUGUGAGUATT | UACUCACAACCUGAAAGCCTT |
| circELMOD3 siRNA2 | CUUUCAGGUUGUGAGUACATT | UGUACUCACAACCUGAAAGTT |
| PCR primer | Sequence | |
|  | Forward | Reverse |
| circELMOD3 | GCTCCAAGTTTGACTGTGCC | GGTGTCCACAGCTTCCCATTA |
| GAPDH | ATCAATGGAAATCCCATCACCA | GACTCCACGACGTACTCAGCG |
| ALDH2 | ATGGCAAGCCCTATGTCATCT | CCGTGGTACTTATCAGCCCA |
| DNASE1L3 | GGACACCACGGTGAAGAAGA | TTGGGAACAACAGAACTGACG |
| STARD5 | CCGGGAAGGCAATGGAGTTT | TCATCCCACTTCACTCGTAGG |
| ACOT12 | TGCTGGAGTTTCCTGCGTTAC | GCATATCCTGTACCATGACCTTG |
