# Supplementary material for: The regulatory role of the circELMOD3-associated ceRNA network in the progression and prognosis of hepatocellular carcinoma
Source: Front Genet. 2025 Apr 15;16:1521360. doi: 10.3389/fgene.2025.1521360 (PMC12037612; doi:10.3389/fgene.2025.1521360)
Supplement: Supplementary file 4 [file DataSheet1.pdf]

Figure S1

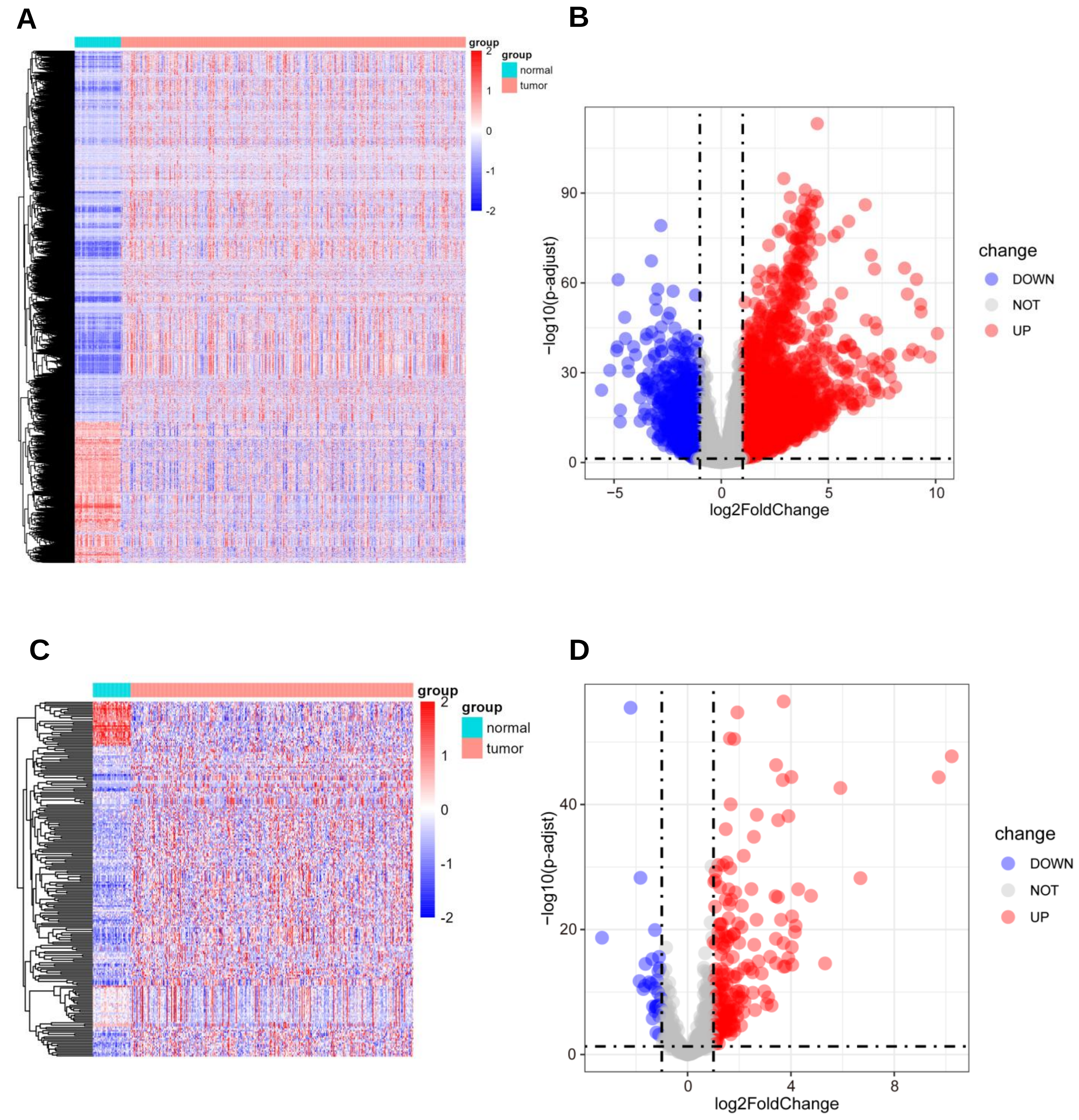

Figure S1

Identification of differentially expressed mRNAs and miRNAs in the TCGA-LIHC. (A, B) Heatmap and volcano plot depicting differentially expressed mRNA in TCGA-LIHC. (C, D) heatmap and volcano plot illustrating differentially expressed miRNA in TCGA-LIHC. Thresholds:|log2FoldChange| > 1, adjusted $p < 0.05$.

Figure S2

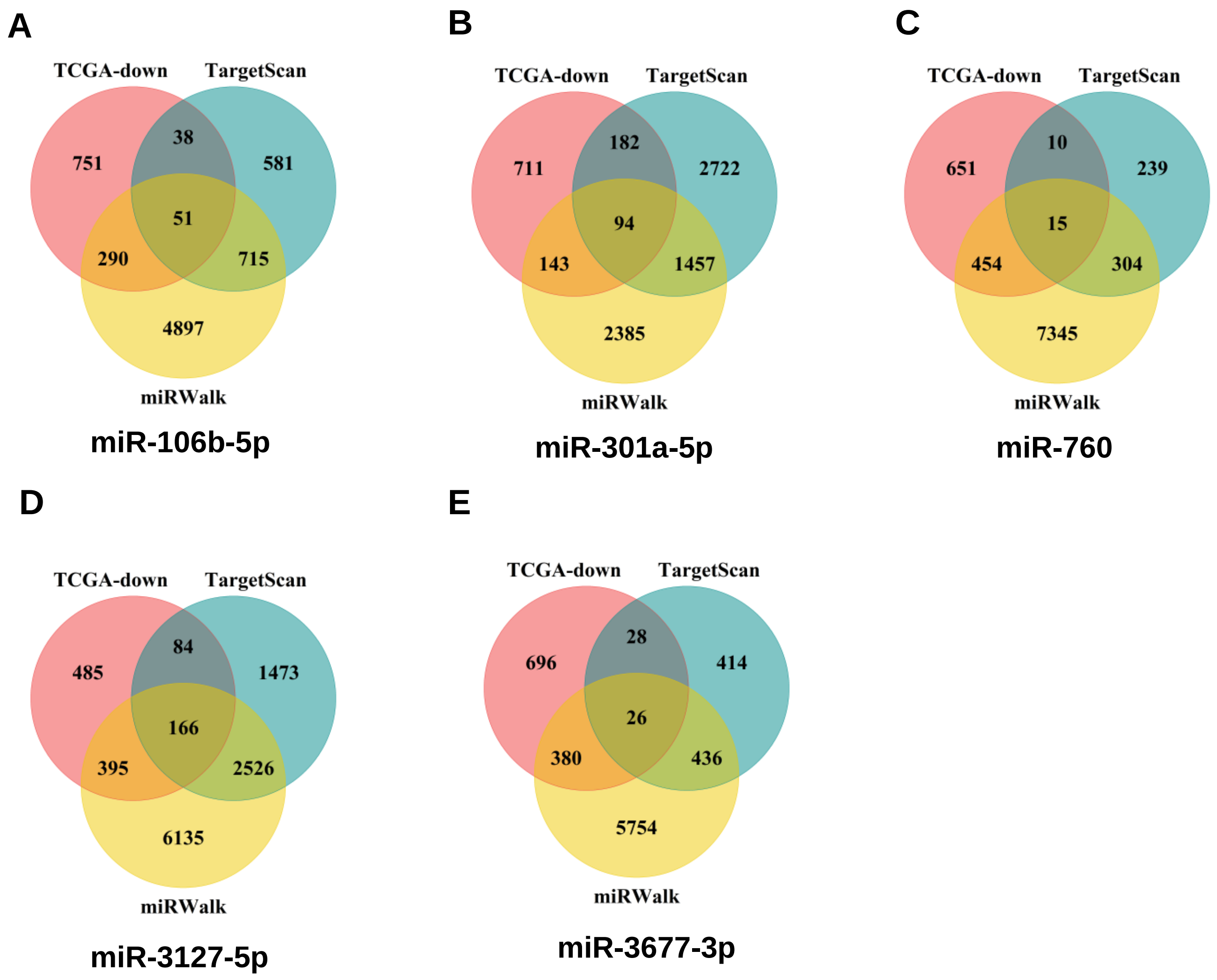

Figure S2

Prediction target mRNA of five miRNAs

Veen diagram analysis of the target mRNA of (A) miR-106b-5p, (B) miR-301a-5p, (C) miR-760, (D) miR-3127-5p and (E) miR-3677-3p and downregulated mRNA in TCGA-LIHC.

Figure S3

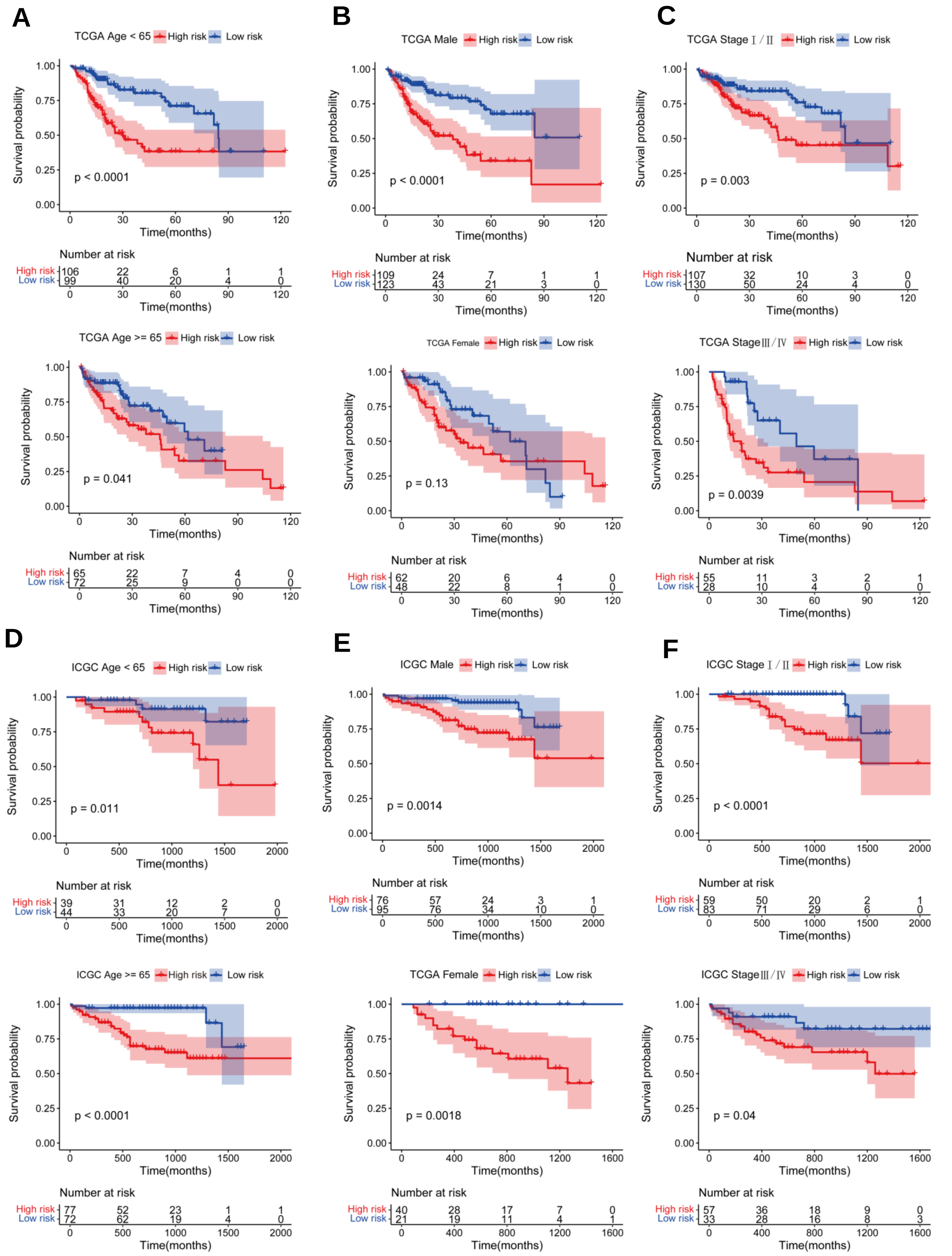

Figure S3

Clinical characteristics correlated with model score.

Kaplan-Meier analysis of HCC patients with different (A) age, (B) Gender and (C) TNM stage in the TCGA-LIHC cohort. Kaplan-Meier plots were drawn for HCC patients with different (D) age, (E) Gender and (F) TNM stage in the LIRI-JP cohort.

Figure S4

A B

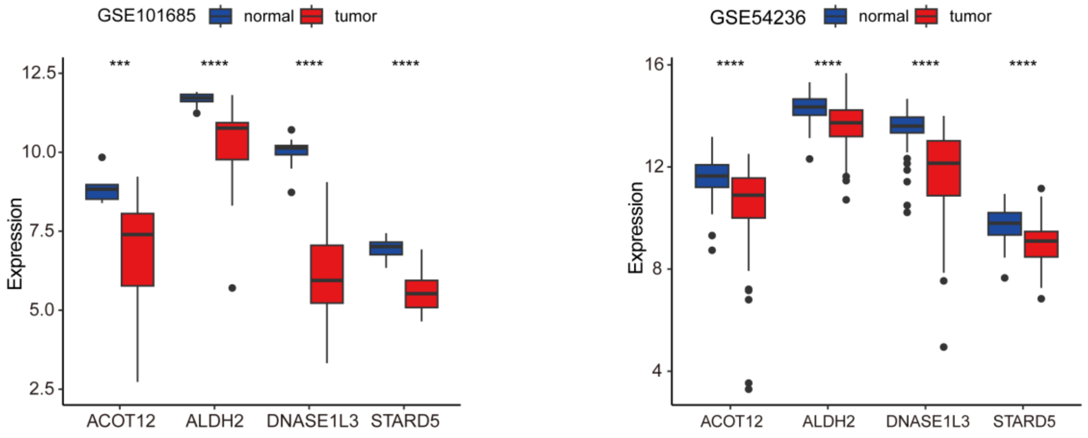

Figure S4
Validation of Key gene expression in GEO database
(A-B) The expression levels of four prognosis genes were validated using GEO database.

Figure S5

Figure 7A Uncropped Western Blots

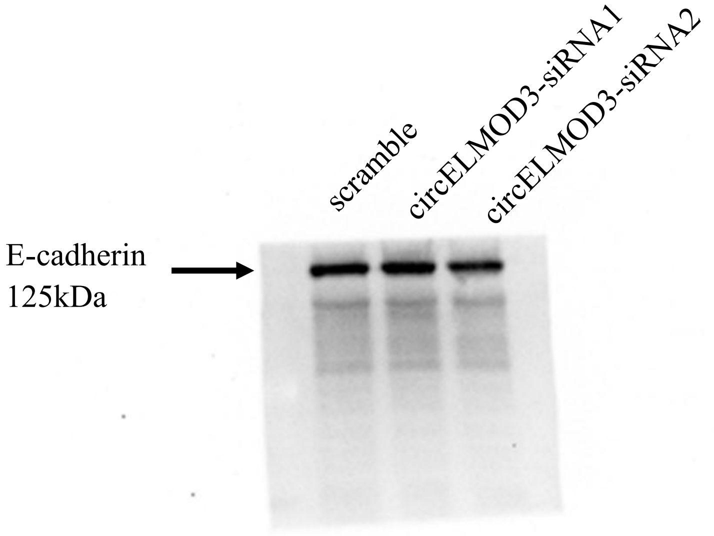

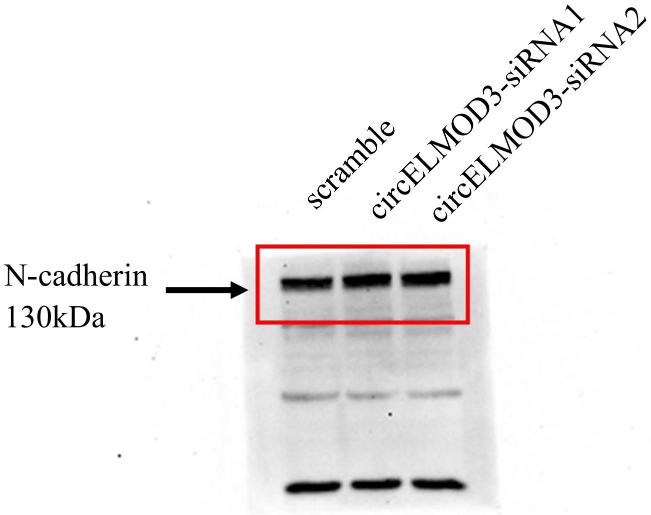

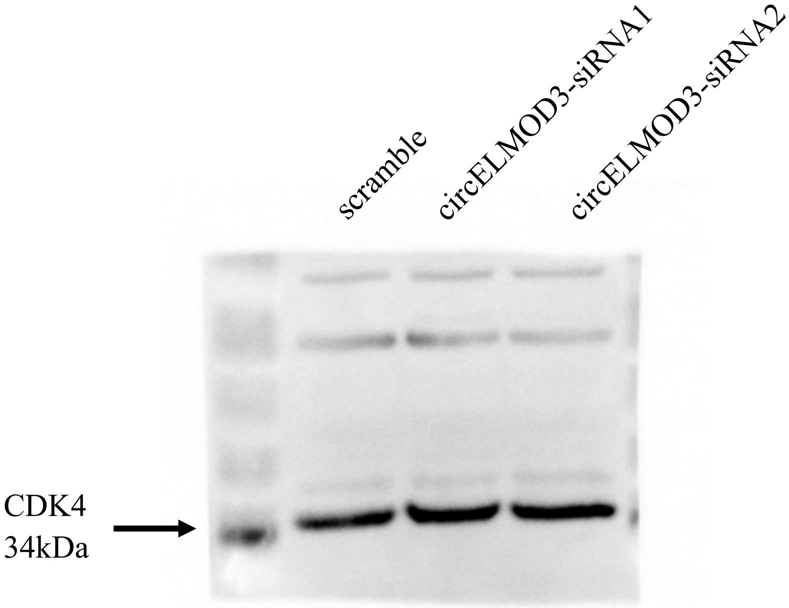

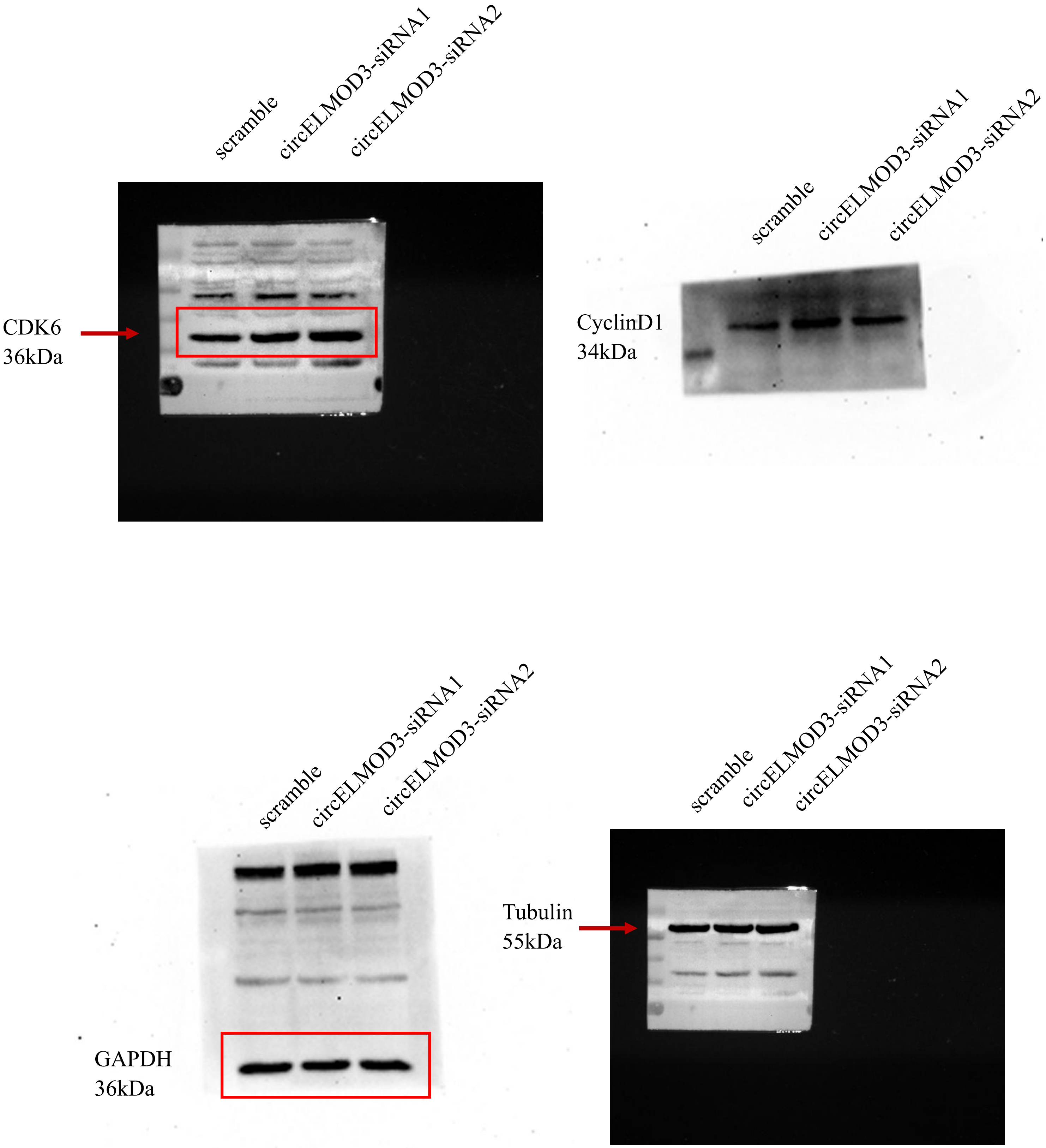
scramble
circELMOD3-siRNA1
circELMOD3-siRNA2
CDK6
36kDa
scramble
circELMOD3-siRNA1
circELMOD3-siRNA2
CyclinD1
34kDa
scramble
circELMOD3-siRNA1
circELMOD3-siRNA2
GAPDH
36kDa
scramble
circELMOD3-siRNA1
circELMOD3-siRNA2
Tubulin
55kDa

Figure 7B Uncropped Western Blots

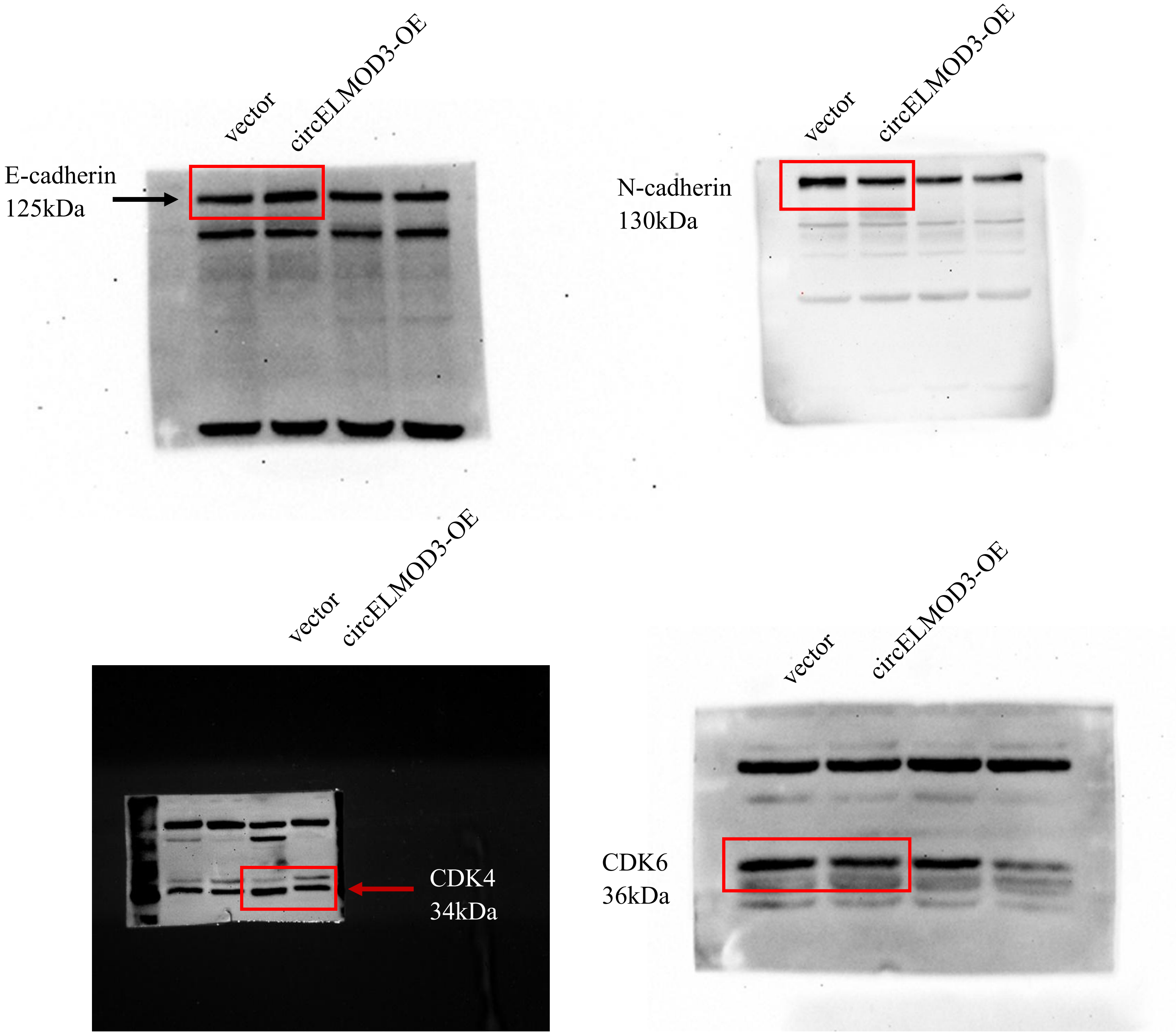

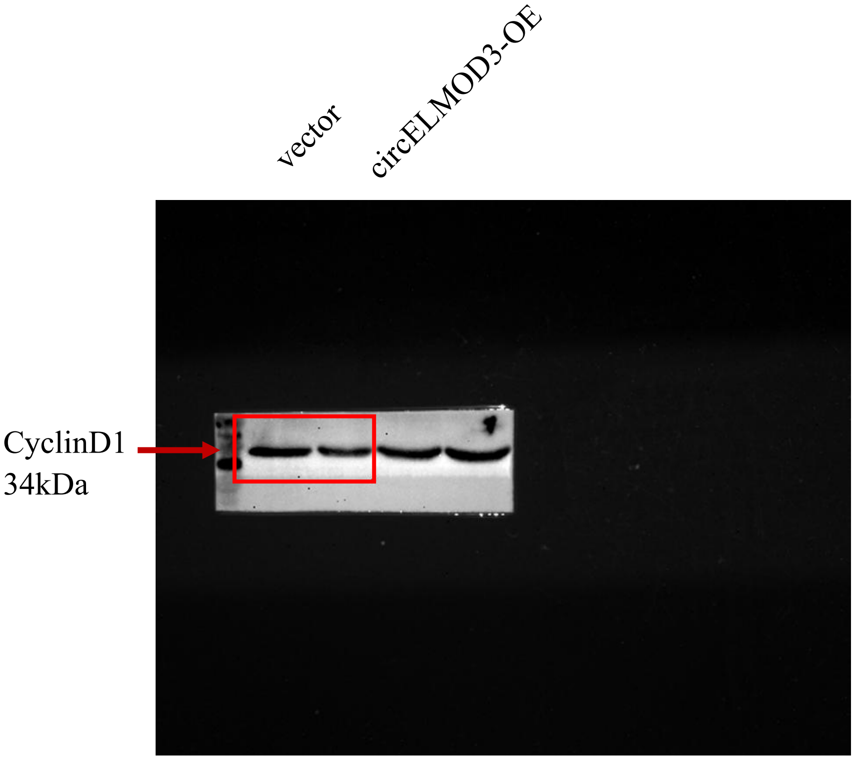
vector
circELMOD3-OE
CyclinD1
34kDa

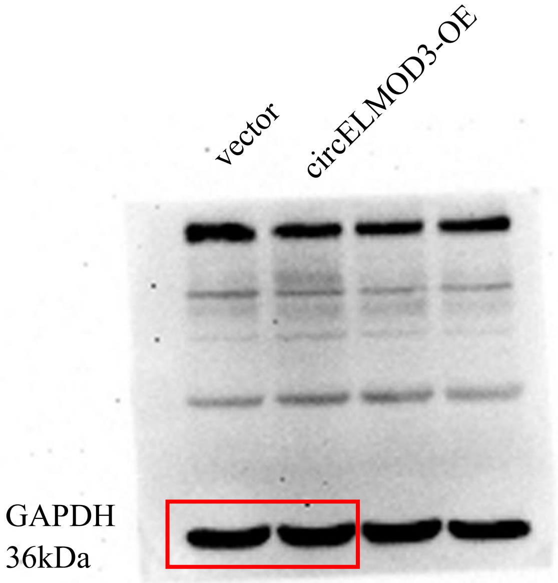
vector
circELMOD3-OE
GAPDH
36kDa

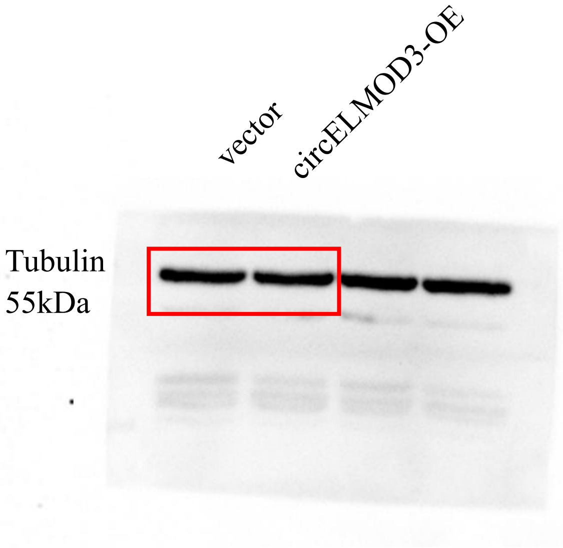
vector
circELMOD3-OE
Tubulin
55kDa
